# Supplementary material for: A fault-intrusion-tolerant system and deadline-aware algorithm for scheduling scientific workflow in the cloud
Source: PeerJ Comput Sci. 2021 Nov 2;7:e747. doi: 10.7717/peerj-cs.747 (PMC8576554; doi:10.7717/peerj-cs.747)
Supplement: Supplemental Information 6 [file peerj-cs-07-747-s006.zip › Myproject1.1/docs/org/cloudbus/cloudsim/VmSchedulerSpaceShared.html]

VmSchedulerSpaceShared


JavaScript is disabled on your browser.


- Overview
- Package
- Class
- Use
- Tree
- Deprecated
- Index
- Help

- Prev Class
- Next Class

- Frames
- No Frames

- All Classes

- Summary:
- Nested |
- Field |
- Constr |
- Method

- Detail:
- Field |
- Constr |
- Method


org.cloudbus.cloudsim

## Class VmSchedulerSpaceShared

- java.lang.Object
- - org.cloudbus.cloudsim.VmScheduler
  - - org.cloudbus.cloudsim.VmSchedulerSpaceShared

- ---

    

  ```
  public class VmSchedulerSpaceShared
  extends VmScheduler
  ```

  VmSchedulerSpaceShared is a VMM allocation policy that allocates one or more Pe to a VM, and
  doesn't allow sharing of PEs. If there is no free PEs to the VM, allocation fails. Free PEs are
  not allocated to VMs

  Since:
  :   CloudSim Toolkit 1.0

- - ### Constructor Summary

    Constructors

    Constructor and Description

    `VmSchedulerSpaceShared(java.util.List<? extends Pe> pelist)`

    Instantiates a new vm scheduler space shared.
  - ### Method Summary

    Methods

    | Modifier and Type | Method and Description |
    | --- | --- |
    | `boolean` | `allocatePesForVm(Vm vm, java.util.List<java.lang.Double> mipsShare)` Allocates PEs for a VM. |
    | `void` | `deallocatePesForVm(Vm vm)` Releases PEs allocated to a VM. |
    | `protected java.util.List<Pe>` | `getFreePes()` Gets the free pes vector. |
    | `protected java.util.Map<java.lang.String,java.util.List<Pe>>` | `getPeAllocationMap()` Gets the pe allocation map. |
    | `protected void` | `setFreePes(java.util.List<Pe> freePes)` Sets the free pes vector. |
    | `protected void` | `setPeAllocationMap(java.util.Map<java.lang.String,java.util.List<Pe>> peAllocationMap)` Sets the pe allocation map. |

    - ### Methods inherited from class org.cloudbus.cloudsim.VmScheduler

      `deallocatePesForAllVms, getAllocatedMipsForVm, getAvailableMips, getMaxAvailableMips, getMipsMap, getPeCapacity, getPeList, getPeMap, getPesAllocatedForVM, getTotalAllocatedMipsForVm, getVmsMigratingIn, getVmsMigratingOut, setAvailableMips, setMipsMap, setPeList, setPeMap, setVmsMigratingIn, setVmsMigratingOut`
    - ### Methods inherited from class java.lang.Object

      `clone, equals, finalize, getClass, hashCode, notify, notifyAll, toString, wait, wait, wait`

- - ### Constructor Detail


    - #### VmSchedulerSpaceShared

      ```
      public VmSchedulerSpaceShared(java.util.List<? extends Pe> pelist)
      ```

      Instantiates a new vm scheduler space shared.

      Parameters:
      :   `pelist` - the pelist
  - ### Method Detail


    - #### allocatePesForVm

      ```
      public boolean allocatePesForVm(Vm vm,
                             java.util.List<java.lang.Double> mipsShare)
      ```

      **Description copied from class: `VmScheduler`**

      Allocates PEs for a VM.

      **Specified by:**
      :   `allocatePesForVm` in class `VmScheduler`

      Parameters:
      :   `vm` - the vm
      :   `mipsShare` - the mips share

      Returns:
      :   $true if this policy allows a new VM in the host, $false otherwise


    - #### deallocatePesForVm

      ```
      public void deallocatePesForVm(Vm vm)
      ```

      **Description copied from class: `VmScheduler`**

      Releases PEs allocated to a VM.

      **Specified by:**
      :   `deallocatePesForVm` in class `VmScheduler`

      Parameters:
      :   `vm` - the vm


    - #### setPeAllocationMap

      ```
      protected void setPeAllocationMap(java.util.Map<java.lang.String,java.util.List<Pe>> peAllocationMap)
      ```

      Sets the pe allocation map.

      Parameters:
      :   `peAllocationMap` - the pe allocation map


    - #### getPeAllocationMap

      ```
      protected java.util.Map<java.lang.String,java.util.List<Pe>> getPeAllocationMap()
      ```

      Gets the pe allocation map.

      Returns:
      :   the pe allocation map


    - #### setFreePes

      ```
      protected void setFreePes(java.util.List<Pe> freePes)
      ```

      Sets the free pes vector.

      Parameters:
      :   `freePes` - the new free pes vector


    - #### getFreePes

      ```
      protected java.util.List<Pe> getFreePes()
      ```

      Gets the free pes vector.

      Returns:
      :   the free pes vector


- Overview
- Package
- Class
- Use
- Tree
- Deprecated
- Index
- Help

- Prev Class
- Next Class

- Frames
- No Frames

- All Classes

- Summary:
- Nested |
- Field |
- Constr |
- Method

- Detail:
- Field |
- Constr |
- Method
